# *Stachys byzantina* K. Koch in the Treatment of Skin Inflammation: A Comprehensive Evaluation of Its Therapeutic Properties

**DOI:** 10.1021/acsomega.4c08830

**Published:** 2024-12-04

**Authors:** José Alisson da Silva Lima, Victor Campana Leite, Jéssica Pereira Silva, Marcelle Andrade Ferrarez, Guilherme Dessupoio Bahia, Luan Vianelo Netto Rezende, Maria Clara Machado Resende Guedes, Gilson Costa Macedo, Natália Prado da Silva, Guilherme Diniz Tavares, Ana Carolina Cruz Reis, Giovanna Oliveira Follis, Vanessa Viana Lempk, Maria Fernanda Fernandes, Elita Scio, Nícolas de Castro Campos Pinto

**Affiliations:** † Laboratory of Bioactive Natural Products, Department of Biochemistry, Institute of Biological Science, Federal University of Juiz de Fora, Juiz de Fora, MG 36036-900, Brazil; ‡ Center for Cellular Technology and Applied Immunology (IMUNOCET), Department of Parasitology, Microbiology and Immunology, Institute of Biological Science, Federal University of Juiz de Fora, Juiz de Fora, MG 36036-900, Brazil; § Laboratory of Nanostructured Systems Development, Department of Pharmaceutical Science, Federal University of Juiz de Fora, Juiz de Fora, MG 36036-900, Brazil

## Abstract

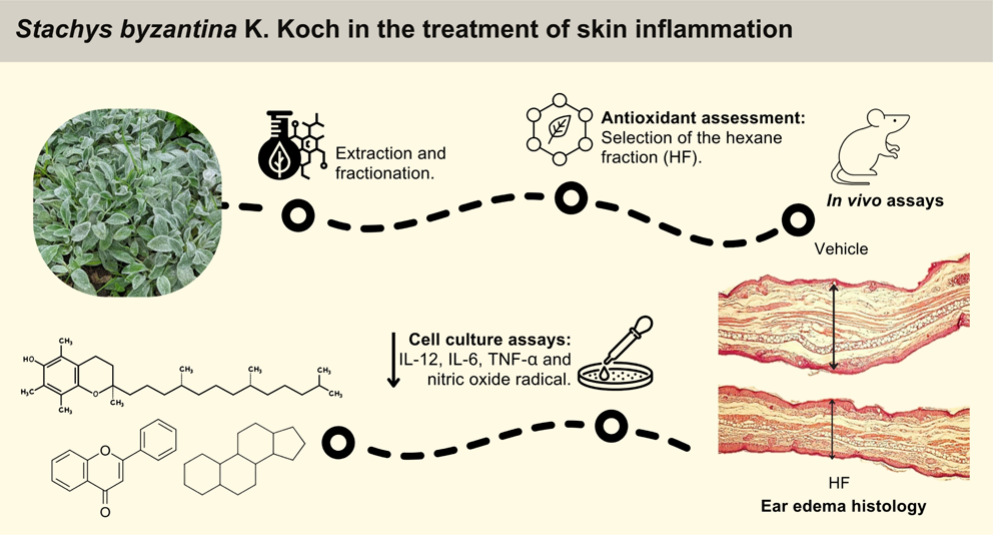

*Stachys byzantina* is a plant widely cultivated for food and medicinal purposes. *Stachys* species have been reported as anti-inflammatory, antibacterial, anxiolytic, and antinephritic agents. This study aimed to evaluate the anti-inflammatory potential of the ethanolic extract (EE) from the aerial parts of *S. byzantina* and its most promising fraction in models of acute and chronic inflammation, including a psoriasis-like mouse model. The EE was fractionated into hexane (HF), dichloromethane (DF), ethyl acetate (AF), and hydroalcoholic (HD) fractions. Screening for anti-inflammatory activity based on nitric oxide inhibition (IC_50_ μg/mL: HF 24.29 ± 5.87, EE 176.45 ± 18.65), hydroxyl radical scavenging (HF 3.89 ± 0.61, EE 6.38 ± 2.25), β-carotene/linoleic acid assay (HF 10.13 ± 3.81, EE 25.64 ± 2.12), and ORAC identified HF as the most active fraction. Topical application of HF effectively reduced croton oil- and phenol-induced ear edema in mice, with no statistical difference to the reference drugs. A formulation containing HF showed significant activity in the imiquimod-induced psoriasis model, reducing pro-inflammatory cytokines and nitric oxide production in macrophages, with no cytotoxicity to skin cells. Phytochemical analysis of HF revealed the presence of terpenes, steroids (491.68 ± 4.75 mg/g), phenols (34.30 ± 4.96 mg/g), flavonoids (151.77 ± 6.66 mg/g), and α-tocopherol, which was identified and quantified by HPLC-UV analysis (10.56 ± 0.97 mg/g of HF). These findings highlight the therapeutic potential of *S. byzantina* for skin inflammation, particularly contact dermatitis and psoriasis, encouraging further studies, including in human volunteers.

## 1 Introduction

The skin plays a fundamental role in protecting the body against environmental harmful stimuli, as the epidermis acts as an effective barrier, preventing the damage induced by exogenous substances.^1^ Inflammatory skin diseases, such as rosacea, acne, atopic dermatitis, and psoriasis, are characterized by intense itching and skin lesions, in addition to telangiectasias, erythema, scaling, and exudation, which may affect the quality of life due to physical discomfort and psychological symptoms.^2^ The generation of reactive oxygen species (ROS) and inflammation are important for skin health maintenance and are minutely controlled by an intricate network of signaling pathways and protein factors.^3,4^ However, if these regulatory processes are affected and the levels of ROS and inflammatory mediators increase, the previously mentioned skin diseases may arise.^4^

Continuous advancement in the development of novel natural bioactives is primordial in the search for new therapies. Innovative approaches for more effective treatments for inflammation are particularly relevant in dermatology, due to the adverse reactions induced by glucocorticoids, which are the main available drugs for this purpose. For this reason, the usage of medicinal plants as active principles to treat skin conditions is increasing.^5^ In this scenario, the global market for natural skin care products will increase from $19.37 billion in 2023 to $21.23 billion in 2024, with a compound annual growth rate (CAGR) of 9.7%. This sector is predicted to reach $31.94 billion by 2028, with a CAGR of 10.8%. Particularly, the psoriasis treatment market is expected to increase from $23.87 billion in 2023 to $26.50 billion in 2024, with a CAGR of 11.0%.^6^

Since the beginning of human civilization, the usage of medicinal plants has been an ordinary practice.^7^ Recent studies emphasize that medicinal plants still play a vital role in people’s daily lives. In addition to acting as supplements, medicinal plants are important alternative agents to contemporary medical treatments, which availability is often limited, promoting health and safety in various communities worldwide. This approach is based on traditional wisdom and observations that are passed down through generations.^8^

In this context, *Stachys* is one of the largest genera belonging to the Lamiaceae family, widely covering Europe, East Asia, and America. *Stachys byzantina* K. Koch, popularly known as hedgenettle or as *peixinho-da-horta* in Brazil, is widely distributed especially in Iraq, Armenia, Iran, and, mainly, Turkey.^9^ However, the genus *Stachys* is also found in the Mediterranean region, southwest Asia, North and South America, and North Africa. Particularly, *S. byzantina* have been cultivated for food purposes and in traditional medicine to treat several diseases, including inflammatory disorders, and it is widely consumed as a wild tea remedy, known as “mountain tea”. Anti-inflammatory, antibacterial, antianxiety, antioxidant, and antinephritic properties have been reported for species of the genus *Stachys*.^10^ In Brazil, the popular use of the aerial parts of *S. byzantina* is widespread, as they are commonly employed for ornamental purposes, in traditional cuisine, and as an infusion to treat several conditions, including lung and stomach disorders, tonsillitis, headache, and other inflammatory processes.^11^ Studies on *S. byzantina* have exploited its antipyretic and antispasmodic properties, as well as its potential to treat abdominal pain and accelerate wound healing. Phenolic compounds, recognized for their diverse biological properties, were found in the extracts of *S. byzantina*.^12^ In addition, caffeic acid, chlorogenic acid, ferulic acid, rutin, apigenin, verbascoside, stigmasterol, β-sitosterol and lawsaritol were identified in the species.^13−16^

In light of the aforementioned and due to the biological properties attributed to the genus *Stachys*, it was hypothesized that *S. byzantina* aerial parts may present anti-inflammatory compounds useful to be incorporated in pharmaceutical products, including for topical usage. For this reason, the present study was conducted, and brings new evidence surrounding the therapeutic application of this plant. Additionally, this work may contribute to the search for new bioactive substances from nature with anti-inflammatory and antipsoriatic potential.

## 2 Methods

### 2.1 Chemicals and Reagents

Absolute ethanol (Dinâmica, São Paulo, SP, Brazil), croton oil from Santa Cruz Biotechnology (Dallas, TX), phosphoric acid from Quimibrás Indústrias Químicas (Cubatão, SP, Brazil), 3-(4,5-dimethylthiazol2-yl)-2,5-diphenyltetrazolium bromide – MTT (Thermo Fischer Scientific, Waltham, MA), spectrophotometer SpectraMax M2 (Molecular Devices, San Jose, CA), and commercial kits for measuring cytokines (Becton & Dickinson Company, Franklin Lakes, NJ) were used in this study. Roswell Park Memorial Medium (RPMI) 1640 and fetal bovine serum were from Gibco Scientific (Waltham, MA). Silica gel 60 F254 TLC plate was acquired from Merck (Darmstadt, HE, Germany). HPLC grade solvents were purchased from Tedia (Fairfield, CO). Water for the HPLC mobile phase was purified in a Milli-Q-plus System (Millipore, Burlington, MA). Sodium nitroprusside, LPS from *Escherichia coli*, trypan blue, sulfanilamide, *N*-(naphthyl)ethylenediamine, β-carotene, 2,2′-azobis2-amidino-propane dihydrochloride were from Sigma-Aldrich (St. Louis, MO). Modik commercial cream (imiquimod) was purchased from Germed (Campinas, SP, Brazil). All other reagents were of the best quality possible.

### 2.2 Plant Material

The aerial parts of *S. byzantina* were collected at the Garden of the Faculty of Pharmacy of Federal University of Juiz de Fora – UFJF, Juiz de Fora, Minas Gerais, Brazil, in March/2022, in the morning (21°77′7419″ S, 43°36′6841″ W). Exsiccate of the plant material was deposited at the Leopoldo Krieger Herbarium (CESJ 46598) for future evidence. This research was registered in the National System for the Management of Genetic Heritage (n◦ A3DD429).

### 2.3 Preparation of the Ethanolic Extract and Fractions

The extraction was carried out using ethanol at 6.7% weight by volume. The plant material was previously dried and reduced to powder, then subjected to ultrasound-assisted extraction for 45 min at 50 °C. The resulting ethanolic extract (EE) was filtered and concentrated until the solvent was completely removed in a rotary evaporator at 40 °C. Subsequently, EE was resuspended in EtOH/H_2_O 8:2 and subjected to fractionation using solvents in increasing order of polarity, resulting in hexane (HF), dichloromethane (DF), ethyl acetate (AF), and hydroethanolic (HD) fractions. These fractions were dried, weighed, and stored under refrigerated conditions, and their yields were calculated. After, EE and fractions were subjected to antioxidant assays to screen the most promising plant derivative.

### 2.4 Antioxidant Assays

#### 2.4.1 Inhibition of the Nitric Oxide (^•^NO) Radical by the Griess Reaction

Briefly, 62.5 μL of EE and fractions or quercetin (reference substance), and sodium nitroprusside were added to a 96-well microplate, followed by incubation for 1h at room temperature. Then, 125 μL of Griess reagent (1% sulfanilamide +0.1% *N*-(naphthyl)ethylenediamine 1:1 in 2.5% phosphoric acid) was added. Phosphate buffer was the negative control. Finally, the absorbance was read at 540 nm.^17^ The tested concentrations were 250, 125, 62.5, 31.25, 15.62, and 7.81 μg/mL in triplicate.

The initial proposal for conducting the nitric oxide radical inhibition assay was to perform a screening between EE and fractions for their anti-inflammatory potential. The most promising plant derivatives were subjected to in-depth studies. For this reason, both HF and EE were selected to further investigations.

#### 2.4.2 β-Carotene/Linoleic Acid Assay

First, an emulsion containing β-carotene and linoleic acid was prepared using Tween 40 and dichloromethane. After mixing, the dichloromethane was removed using nitrogen gas, and oxygen-saturated water was added. The absorbance of the emulsion was adjusted between 0.6 and 0.7 at 470 nm. In a 96-well plate, 250 μL of this emulsion and 10 μL of EE, HF, or quercetin (reference substance) were added. Absorbance readings were taken immediately after preparation and after 120 min, both at 45 °C. Methanol was used as the negative control.^18^ The tested concentrations were 38.46, 19.23, 9.61, 4.8, 2.4, and 1.2 μg/mL in triplicate.

#### 2.4.3 Scavenging of the Hydroxyl Radical (^•^OH)

Briefly, 100 μL of EE, HF, and gallic acid (reference substance) were added to test tubes, followed by 100 μL of 2-deoxyribose 5 mM, 3% hydrogen peroxide at 100 mM, and iron sulfate 6 mM. The mixture was incubated for 15 min at room temperature. After, 500 μL of phosphoric acid and thiobarbituric acid was added. Subsequently, all tubes were heated in a water bath for 15 min at 98 °C. Phosphate buffer was used as the negative control. The absorbance was read in a 96-well microplate at 532 nm.^19^ The tested concentrations were 200, 166.6, 133.3, 100, 66.6, and 33.3 μg/mL in triplicate.

#### 2.4.4 Oxygen Radical Absorbance Capacity Assay (ORAC)

Two hundred microliters of water were added to the peripheral wells of a 96-well microplate. Cyclodextrin was used for better solubility. Then, EE, HF, and Trolox (reference substance) 25 μL at 6.25 μg/mL (final concentration) and 150 μL fluorescein at 40 nM were added in triplicate, followed by incubation at 37 °C for 30 min. Subsequently, AAPH (2,2′-azobis2-amidino-propane dihydrochloride) 25 μL was added, followed by homogenization for 10 min. Cyclodextrin was used as the negative control. Kinetic readings were taken every 10 min using a fluorescence spectrophotometer with an excitation filter at 485 nm and emission at 538 nm.^20^

Based on HF most promising response in antioxidant assays, this plant derivative was selected for further investigation and a more comprehensive assessment of its properties and therapeutic potential.

### 2.5 *In Vitro* Assessment of Cell Viability/Cytotoxicity and Measurements of Inflammatory Markers

#### 2.5.1 Cell Line and Cell Culture Conditions

J774A.1 lineage macrophages (ATCC TIB-67), L929 fibroblast (ATCC CCL-1 NCTC), and HaCaT keratinocytes (ATCC CRL-2309) were used for HF cellular toxicity evaluation. The cells were maintained in 75 cm^3^ culture flasks with Dulbecco’s Modified Eagle Medium (DMEM), supplemented with 10% fetal bovine serum (FBS), 1% antibiotic, and cultured in a humidified incubator at 37 °C, 5% CO_2_. Cells were cultured until approximately 80% confluence. Subsequently, the medium was removed, and the cells were washed with phosphate-buffered saline (PBS) and detached by scrapping. Afterward, 5 mL of DMEM medium supplemented with 10% FBS and 1% antibiotic solution was added for enzyme inactivation. The cell solution was transferred to a conical tube and centrifuged for 4 min at 3500 rpm. Then, the supernatant was discarded, and the cells were resuspended in the same solution. After the procedure, the cells were subjected to the subsequent experiments.

#### 2.5.2 Cell Viability Assay

To evaluate HF cytotoxicity, the MTT colorimetric method (3-[4,5-dimethylthiazol-2-yl]-2,5-diphenyltetrazolium bromide) was used. J774A.1 cells were seeded in 96-well microplates at a concentration of 5 × 10^4^ cells/well, whereas L929 and HaCaT cell lines were plated at 1,000 cells/well.^21^ The cells were incubated for 24 h at 37 °C in a CO_2_ atmosphere. After incubation, the culture medium was removed and 100 μL of DMEM (10% FBS and 1% antibiotic) containing HF previously solubilized at 1.0 mg/20 μL in DMSO were added for cell lines L929 and HaCaT. For J774A.1, HF was diluted in acetone. The HF concentrations tested were 12.5–100 μg/mL. DMEM medium was used as a negative control. The plates were incubated for another 24 h at 37 °C with 5% CO_2_. After this period, the medium was removed and 90 μL of DMEM supplemented with 10 μL of MTT solution at 5 mg/mL were added, followed by incubation for 2 h and 30 min at 37 °C with 5% CO_2_. Subsequently, the precipitate was dissolved in DMSO 100 μL and the absorbance was read at 595 nm. The determination of the percentage of cellular viability was obtained by averaging the absorbances in triplicate, with the average absorption value of the negative control group considered as 100% viability.

#### 2.5.3 *In Vitro* Cytokine Inhibition

To determine cytokine production, J774A.1 cells were cultured and plated as previously described. Then, the cells were stimulated with 1 μg/mL of *E. coli* LPS and incubated for 1h (37 °C, 5% CO_2_). After incubation, the cells were treated with HF 50 μL at 125; 62.5; 31.25; and 15.6 μg/mL per well in a microplate. After 48 h, the supernatant was collected and the cytokines levels were determined by sandwich-type ELISA using commercial kits, according to the manufacturer protocol (BD Biosciences, Franklin Lakes, NJ). LPS was used as a negative control. The assay was carried out in quadruplicate and repeated at least twice. The results were expressed in pg/mL.

#### 2.5.4 ^•^NO Radical Dosage in Cell Culture

To determine the concentration of NO, the concentration of nitrite (NO_2_^–^) in the cell culture supernatant was measured using the Griess reaction. J774A.1 cells were plated as previously described and stimulated with LPS 1.0 μg/mL together with interferon-γ (IFN-γ) 0.9 ng/mL. After 60 min, the cells were treated with HF 50 μL at 125; 62.5; 31.25; and 15.6 μg/mL per well. After 48 h of incubation, the supernatants were collected and the nitrite concentration was determined as follows: in a 96-well flat-bottom plate, 50 μL per well of culture supernatants was added, followed by 30 μL of Griess reagent. After 10 min of incubation at room temperature, absorbances were read at 540 nm. The amount of nitrite (NO_2_) was calculated using a standard curve of a sodium nitrite (NaNO_2_) solution. LPS and IFN-γ together, with no treatments, were used as the negative control. The assay was performed in quadruplicate and the results were expressed in μM.^22^

### 2.6 Anti-inflammatory Trials in Mice

#### 2.6.1 Animals

For the assessment of acute topical anti-inflammatory activity, male Swiss mice aged 30 days, weighing between 25 and 30 g, were used. For the antipsoriatic activity, Balb/C male mice aged 60 days, weighing between 20 and 30 g, were employed. The animals were provided by the UFJF Center for Reproductive Biology (CBR) and were housed in cages under controlled temperature (22 °C) and light cycle, with unrestricted access to water and standard rodent chow. Each experimental group consisted of 7–8 animals. All procedures and experimental protocols adhered to the Ethical Principles in Animal Experimentation established by the National Council for the Control of Animal Experimentation (CONCEA) and were approved by UFJF Animal Ethics Committee (CEUA), under protocol numbers 013/2022 and 038/2022.

#### 2.6.2 Topical Acute Anti-inflammatory Activity

##### 2.6.2.1 Croton Oil-Induced Ear Edema Test

Twenty microliters of a fresh solution containing 2.5% (v/v) croton oil diluted in acetone were topically administered to the right ear pinna, and an equal volume of acetone was applied to the left ear pinna of each animal. Immediately, the animals received topical administration of HF 20 μL at different concentrations (0.1, 0.5, and 1.0 mg/20 μL), dexamethasone 0.1 mg/20 μL (reference substance), or acetone (vehicle). All the treatments were applied exclusively to the right ear pinna. After 4 h, animals were euthanized, and identical ear fragments with 6 mm diameter were obtained from both ears of each animal using a metal punch. The ear fragments were subsequently weighed on an analytical balance and the weight difference between them indicated the edema intensity.^23^

##### 2.6.2.2 Phenol-Induced Ear Edema

First, 20 μL of a fresh solution containing phenol 10% (v/v) diluted in acetone was topically administered to the right ear pinna, and an equal volume of acetone was applied to the left ear pinna of each animal. Immediately, the animals received topical administration of HF 20 μL at 1.0 mg/20 μL, dexamethasone at 0.1 mg/20 μL (reference substance), or acetone (vehicle). The edema intensity was measured 1 h after the induction of the inflammatory process,^24^ following the same procedure detailed in Section 2.6.2.1.

#### 2.6.3 Topical Chronic Anti-inflammatory Activity

##### 2.6.3.1 Imiquimod-Induced Ear Edema

HF antipsoriatic and chronic anti-inflammatory efficacy were assessed at the same time, using the imiquimod-induced ear edema test^25^ with some modifications. From Day 1 to Day 5, 3 mg of commercial imiquimod cream (Modik at 50 mg/g) was topically applied once daily to the inner ear pinna of each animal to induce a cutaneous inflammatory process similar to psoriasis. Starting from Day 6, 10 mg of the vehicle (pharmaceutical formulation containing 5% emulsifier, 5% humectant, 3% emollient, 1% antioxidant, and 0.7% preservative agents, in addition to water q.s.), HF 6% (w/w), HF 12% (w/w), and clobetasol 0.5 mg/g (control) were topically applied twice daily (12/12h) for 5 consecutive days to the mice right inner ear pinna. All treatments were incorporated into the same pharmaceutical formulation described above. Additionally, 10 mg of vehicle was topically applied to the left ear. On Day 10, the animals were euthanized and a 6 mm diameter fragment was obtained from both ears of each animal using a metal punch, weighed on an analytical balance, and subjected to histopathological analysis. The weight difference between the ears indicated the intensity of the edema and, consequently, of the inflammatory process. The HF concentrations in pharmaceutical formulations were chosen to maintain at least the same percentage (w/w) of HF 1.0 mg/20 μL solution prepared in acetone for the acute anti-inflammatory *in vivo* tests, taking into consideration the acetone density. The concentration of clobetasol was the same as that used in clinical practice to treat psoriasis.

##### 2.6.3.2 Histopathological Analysis

Ear biopsies were initially fixed in 70% ethanol for 24 h and subsequently preserved in 10% formalin. Then, the ears were subjected to a dehydration process, paraffin inclusion, and cross-section using a microtome (4 μm). The resulting cross sections were stained with hematoxylin and eosin to assess the presence and intensity of edema, vasodilation, and leukocyte infiltration. Representative areas were selected for qualitative analysis by optical microscopy, with images captured by Image-Pro Plus 6.0 software at magnifications of 40, 100, and 400×. Additionally, quantitative analyses of the edema thickness were conducted using the same software, with 100 μm field apertures. These analyses were carried out at five different points of each biopsy (*n* = 8) in triplicate, totaling 120 measurements for each group. The analyzed points in each fragment were at a distance of 250 μm from each other.

### 2.7 Phytochemical Evaluation

#### 2.7.1 Thin-Layer Chromatography (TLC)

HF preliminary phytochemical analysis was performed using spray dyeing reagents for thin-layer chromatography (TLC).^26^ Plates coated with silica gel 60 F254 were used, and 10 μL (4 mg/mL) of HF was applied. A mobile phase consisting of hexane/ethyl acetate 7:3 (v/v) was used. After eluent evaporation, the plates were sprayed with specific solutions (Lieberman*n*-Burchard, NP/PEG, Dragendorff, vanillin sulfuric, KOH 5%, and FeCl_3_ 1%) to identify different classes of secondary metabolites.

#### 2.7.2 Total Triterpenes and Steroid Content

In test tubes, HF 100 μL (1000 μg/mL) solubilized in chloroform was transferred and the solution was heated in a water bath until the solvent was completely dry. Afterward, 250 μL of vanillin and 500 μL of sulfuric acid were added and heated at 60 °C for 30 min. Then, in an ice bath, 2500 μL of acetic acid was added to the tubes. Upon reaching room temperature, the mixtures were homogenized and subjected to spectrophotometric analysis at 548 nm. The same procedure was performed for the β-sitosterol standard curve.^27^

#### 2.7.3 Total Phenolic Content

The Folin–Ciocalteu method was employed to determine HF phenolic content.^28^ In a 96-well microplate, 120 μL of the Folin–Ciocalteu solution (20% v/v in water), 100 μL of sodium carbonate (4% w/v in water), and 30 μL of HF (500 μg/mL) were added. Then, the microplate was kept in the dark for 30 min and the absorbance was read at 770 nm in triplicate. The same procedure was carried out for the tannic acid standard curve (reference substance).

#### 2.7.4 Total Flavonoid Content

In a 96-well microplate, HF 40 μL (1000 μg/mL), aluminum chloride diluted in ethanol 40 μL (5% w/v) with 5% acetic acid, and 120 μL of ethanol were added. After the microplate was kept in the dark for 40 min, the absorbance was read at 415 nm in triplicate. The same procedure was carried out for the quercetin standard curve (reference substance).^29^

#### 2.7.5 High-Performance Liquid Chromatography Analysis (HPLC-DAD)

The HPLC analysis was conducted using an Agilent Technologies 1200 Series instrument (Santa Clara, CA) equipped with an automatic injector, quaternary pump, Agilent Eclipse Plus C18 column, and a DAD-UV detector. For HF analysis, an isocratic mobile phase of methanol and UHQ water (98:2, v/v) was used, with a flow rate of 1.3 mL/min. HF was prepared at 1 mg/mL diluted in the mobile phase and injected at 20 μL in triplicate. The UV detection was accomplished at 205 nm. α-tocopherol was identified and quantified by coelution with standard, used at 1, 2, 5, 10, and 20 μg/mL (*r*^2^ = 0.9927).^30^

### 2.8 Statistical Analysis

The tests to evaluate antioxidant and anti-inflammatory activities presented the results as mean ± standard error (s.e.m.). Antioxidant tests were conducted in triplicate, at different concentrations, and one-way ANOVA followed by the Tukey test was applied, except for the ORAC assay, in which two-way ANOVA followed by the Bonferroni test was used. One-way ANOVA was used for *in vivo* anti-inflammatory tests, followed by the Newman-Keuls test. For cell culture assay, one-way ANOVA was performed and the Dunnet test was applied as posthoc. Differences between means were considered significant when *p < 0.05, **p < 0.01, ***p < 0.001 and ****p < 0.0001. The GraphPad Prism 8.0.1 software was used to perform the statistical analyses.

## 3 Results

### 3.1 Antioxidant Evaluation

**Figure 1:**
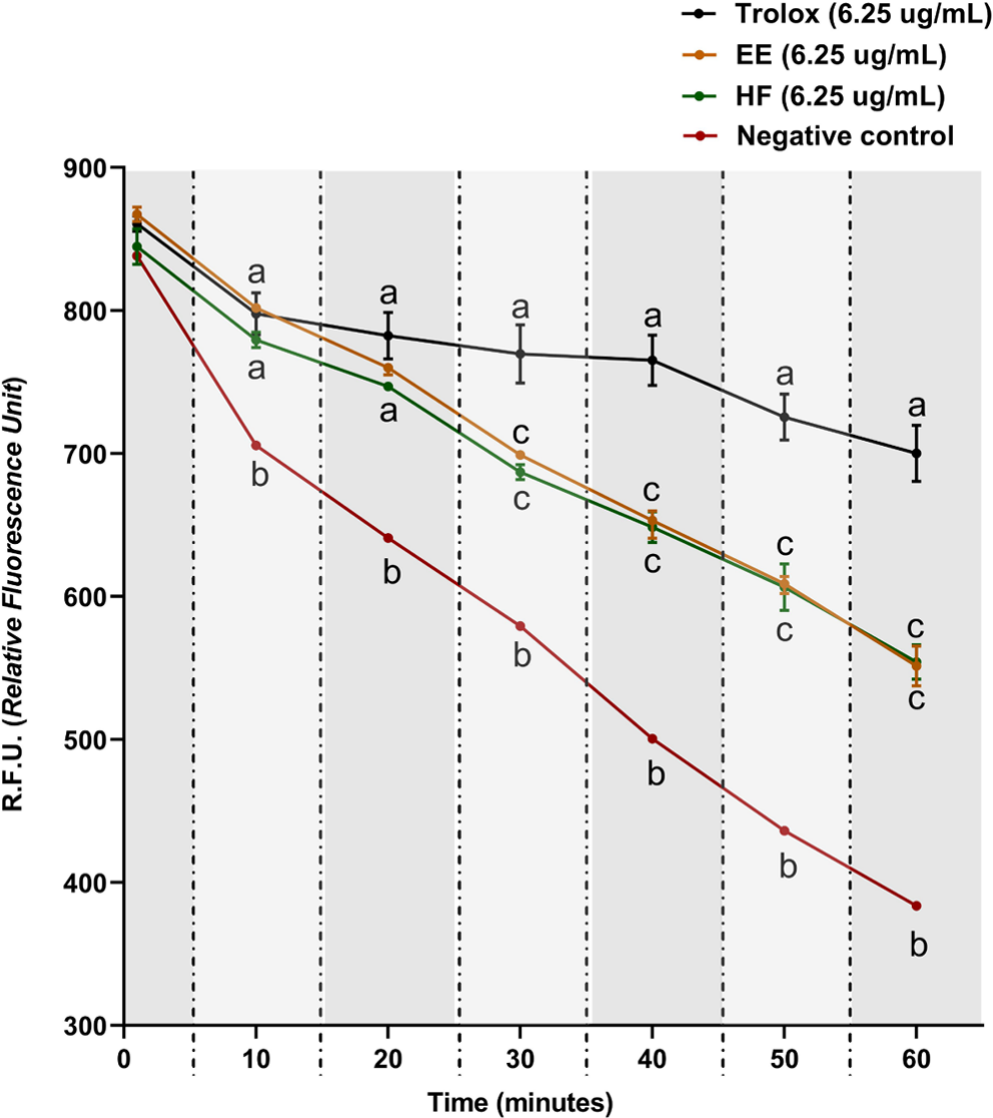
HF and EE kinetics in ORAC assay. Two-way ANOVA followed by Bonferroni test. The values obtained represent the mean ± s.e.m. of R.F.U. (Relative Fluorescence Unit). The data were obtained by six readings, each one being carried out within 10 min. Equal letters indicate no statistical difference (*p* < 0.05).

Fluorescence measurements were achieved for Trolox, EE, and HF to evaluate and compare their capacity to prevent the action of peroxyl and alkoxyl radicals. After six readings over 60 min, EE and HF showed a statistical difference compared to Trolox after 30 min. Both plant derivatives were quite similar. All the sample groups were statistically different compared to the negative control in all time points (Figure 1).

EE and HF showed the lowest IC_50_ values in the nitric oxide radical inhibition assay; however, there was a statistical difference between both. In contrast, HF and quercetin showed no statistical differences. The remaining fractions were unable to achieve an inhibition percentage greater than 50% at the highest concentration tested. HF, EE, and quercetin showed statistical differences in the β-carotene/linoleic acid assay; however, HF presented a lower IC_50_ value compared to EE. Furthermore, in the ^•^OH radical inhibition assay, HF demonstrated a lower IC_50_ compared to EE, although no statistical differences were observed between them (Table 1).

**Table 1:** IC_50_ Values for EE and Fractions in Antioxidant Assays^a^

**IC**_**50**_**(μg/mL)**			
**sample/reference**	**inhibition of nitric oxide (^•^NO) radical**	**β-carotene/linoleic acid assay**	**scavenging of hydroxyl radical (^•^OH)**
**quercetin**	25.68 ± 3.45^**a**^	3.38 ± 0.70^**a**^	N/A
**gallic acid**	N/A	N/A	0.99 ± 0.38^**a**^
**HF**	24.29 ± 5.87^**a**^	10.13 ± 3.81^**b**^	3.89 ± 0.61^**ab**^
**EE**	176.45 ± 18.65^**b**^	25.64 ± 2.12^**c**^	6.38 ± 2.25^**b**^
**DF**	>250	N/A	N/A
**AF**	>250	N/A	N/A
**HD**	>250	N/A	N/A

a ANOVA followed by the Tukey test. The values obtained represent the mean ± s.e.m. in triplicate. Equal letters indicate no statistical difference between the groups (*p* < 0.05). N/A: not available. HF: hexane fraction, EE: ethanolic extract, DF: dichloromethane fraction, AF: ethyl acetate fraction and HD: hydroalcoholic fraction.

### 3.2 *In Vitro* Assessment of Cell Viability/Cytotoxicity and Measurements of Inflammatory Markers

**Figure 2:**
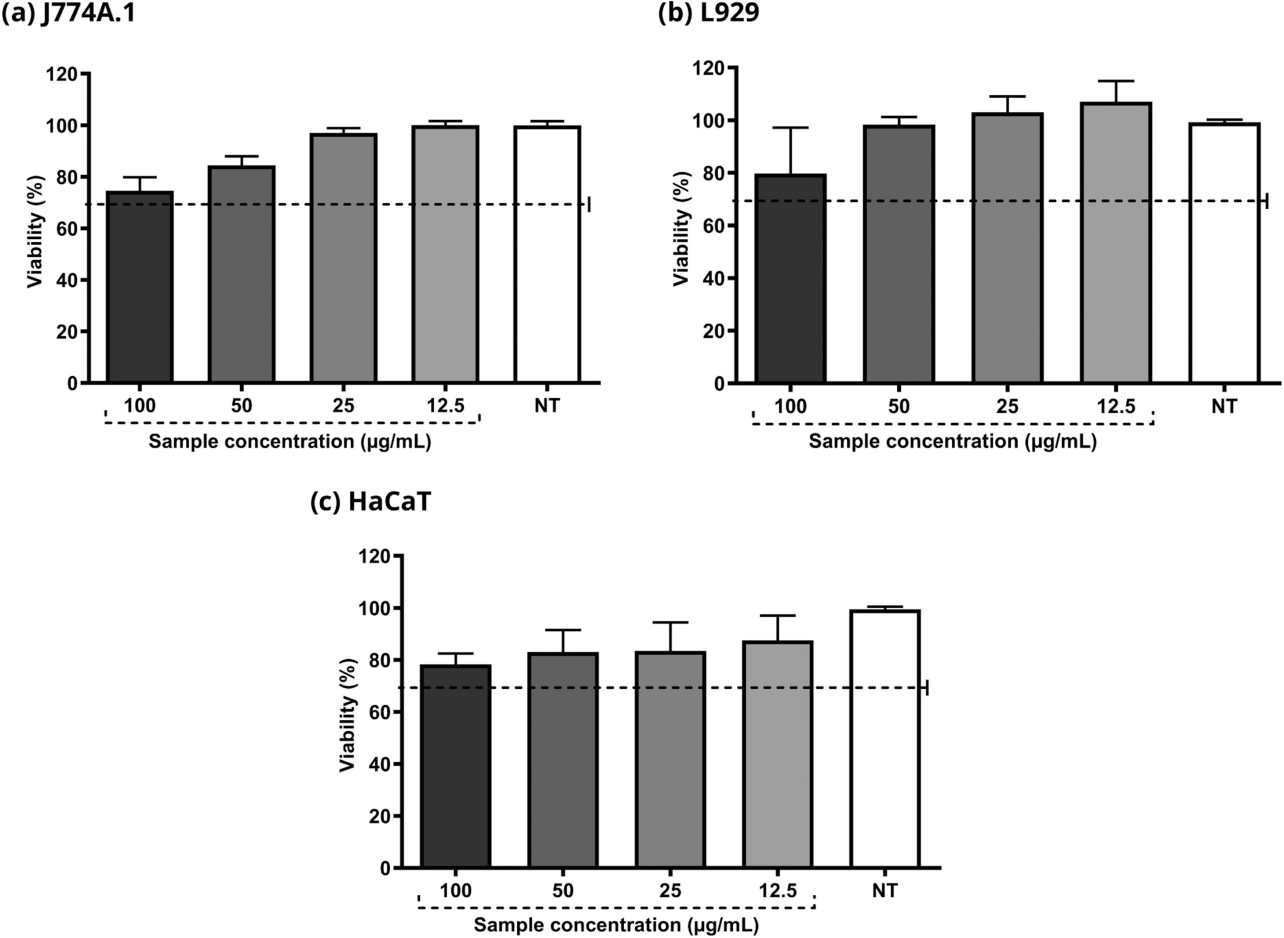
Evaluation of HF cytotoxicity in J774A.1, L929 and HaCaT cells. The concentrations used varied between 12.5 and 100 μg/mL. DMEM culture medium was used as the negative control. Viability above 70% characterizes a nontoxic sample.

The cells maintained a viability greater than 70% in all the experiments carried out at different concentrations in J774A.1 (macrophages), L929 (fibroblasts), and HaCaT (keratinocytes) cells. These results suggest that HF does not present relevant toxicity for these particular skin cells (Figure 2).

**Figure 3:**
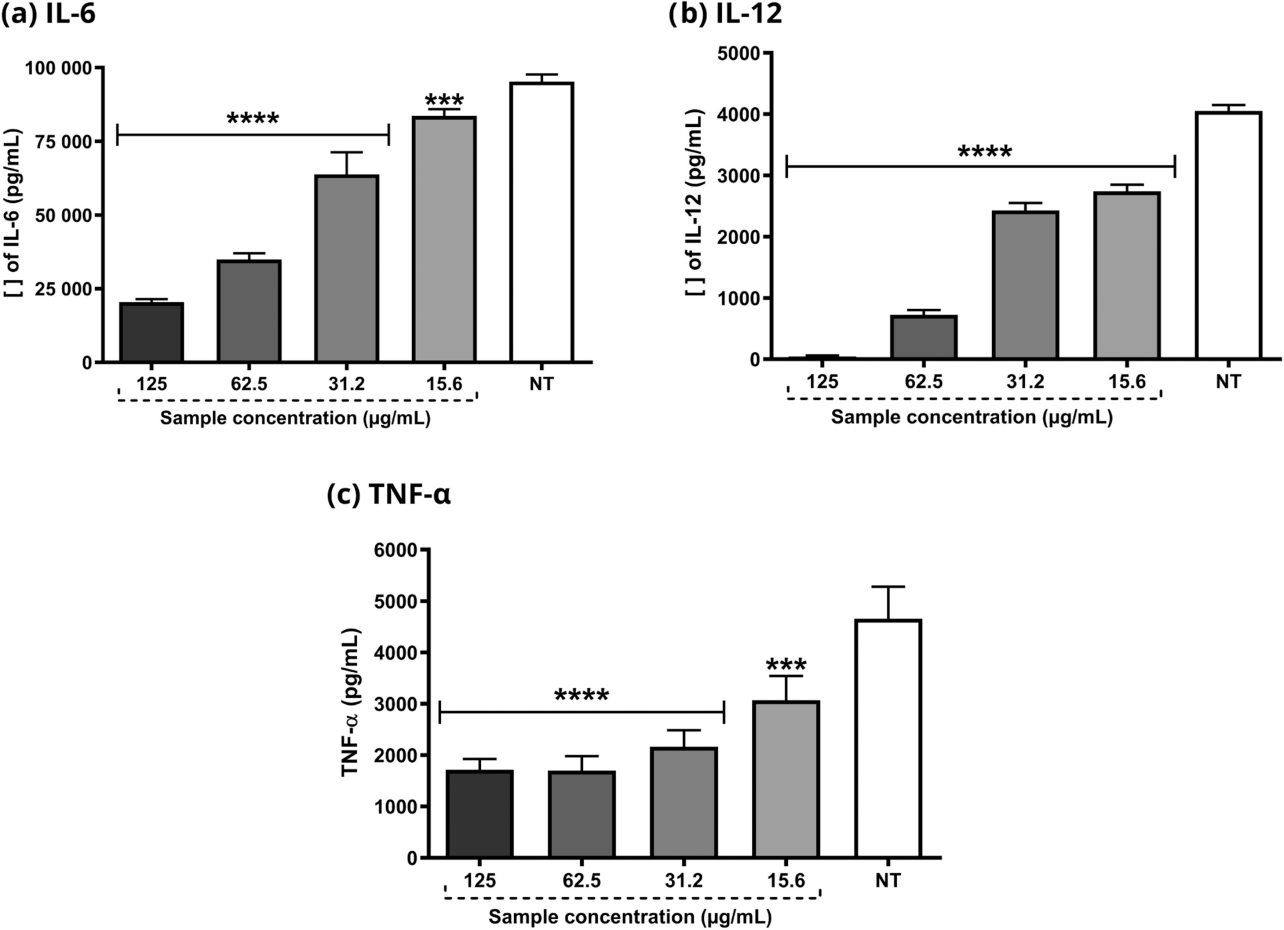
Determination of IL-6, IL12, and TNF-α cytokines in J774A.1 cell line stimulated with *E. coli* LPS. HF concentrations range from 125 to 15.6 μg/mL. ANOVA followed by the Dunnet test. Significant values: ****p < 0.0001 and ***p < 0.001.

As shown in Figure 3, HF also reduced the levels of the pro-inflammatory cytokines IL-6, IL-12, and TNF-α at all concentrations tested in cells.

**Figure 4:**
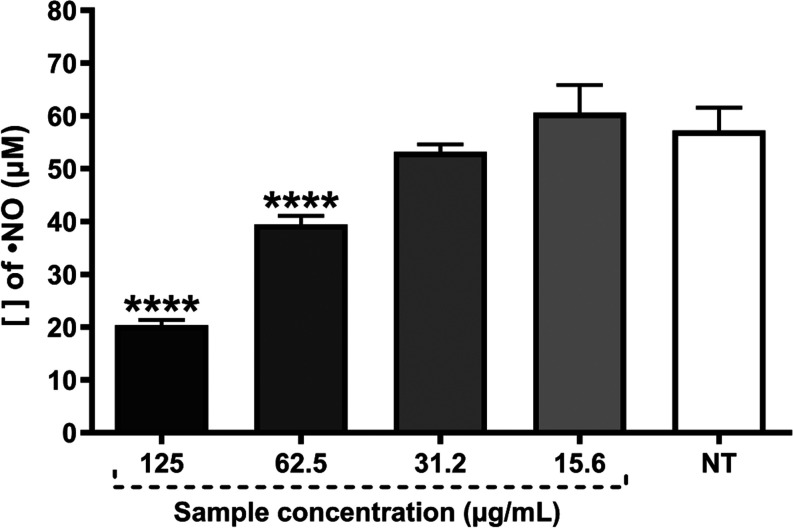
Determination of ^•^NO radical concentration in J774A.1 cell line stimulated with LPS 3 from *E. coli* and IFN-γ. HF concentrations ranged from 125 to 15.6 μg/mL. ANOVA 4 followed by the Dunnet test. Significant values: ****p < 0.0001.

HF 125 and 62.5 μg/mL were able to reduce nitric oxide production by LPS and IFN- γ -stimulated macrophages. Other concentrations did not show any statistical difference compared to the untreated group (Figure 4).

### 3.3 Anti-inflammatory *In Vivo* Trials

#### 3.3.1 Topical Acute Anti-inflammatory Activity

**Figure 5:**
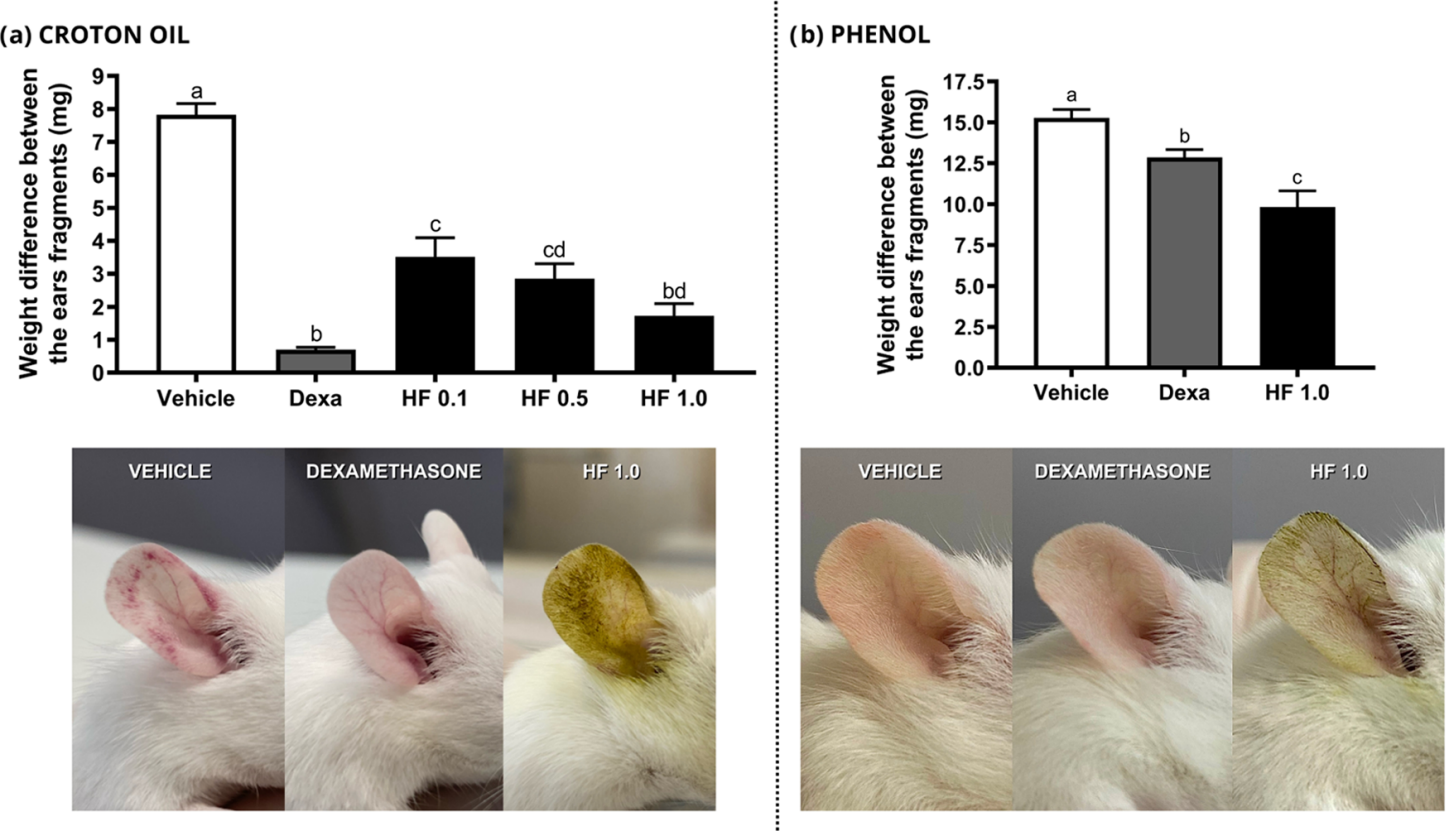
Effect of HF on the inflammatory stimulus induced by croton oil and phenol, in addition to representative photographs of the clinical appearance of mice ears (*n* = 7 and 8 animals, respectively). Negative control (vehicle - acetone), dexamethasone (Dexa) 0.1 mg/20 μL and HF at 0.1; 0.5 and 1.0 mg/20 μL were topically administered immediately after topical application of 2.5% croton oil and phenol 10% (v/v). Values in each column represent the mean ± s.e.m. of the weight difference between ear fragments (mg). ANOVA, followed by the Newman-Keuls test. Equal letters indicate no statistical difference (*p* < 0.05).

Topical application of HF at three different doses reduced the ear edema induced by croton oil. The activity for HF 1.0 mg/20 μL was the most relevant, as the edema was reduced by 78%, and no statistical difference to dexamethasone was found, used as the reference substance (91%). HF 0.5 mg/20 μL and 0.1 mg/20 μL reduced the ear edema by 63% and 55%, respectively (Figure 5a). As HF 1.0 mg/20 μL showed the most promising anti-inflammatory response, this dose was also tested in a further experiment using phenol as a phlogistic agent.

As seen in Figure 5b, HF 1.0 mg/20 μL decreased the phenol-induced ear edema by 36%. This activity was more prominent compared to dexamethasone, which reduced the ear edema by 16%.

#### 3.3.2 Topical Anti-inflammatory Activity in Psoriasis-like Chronic Inflammation

**Figure 6:**
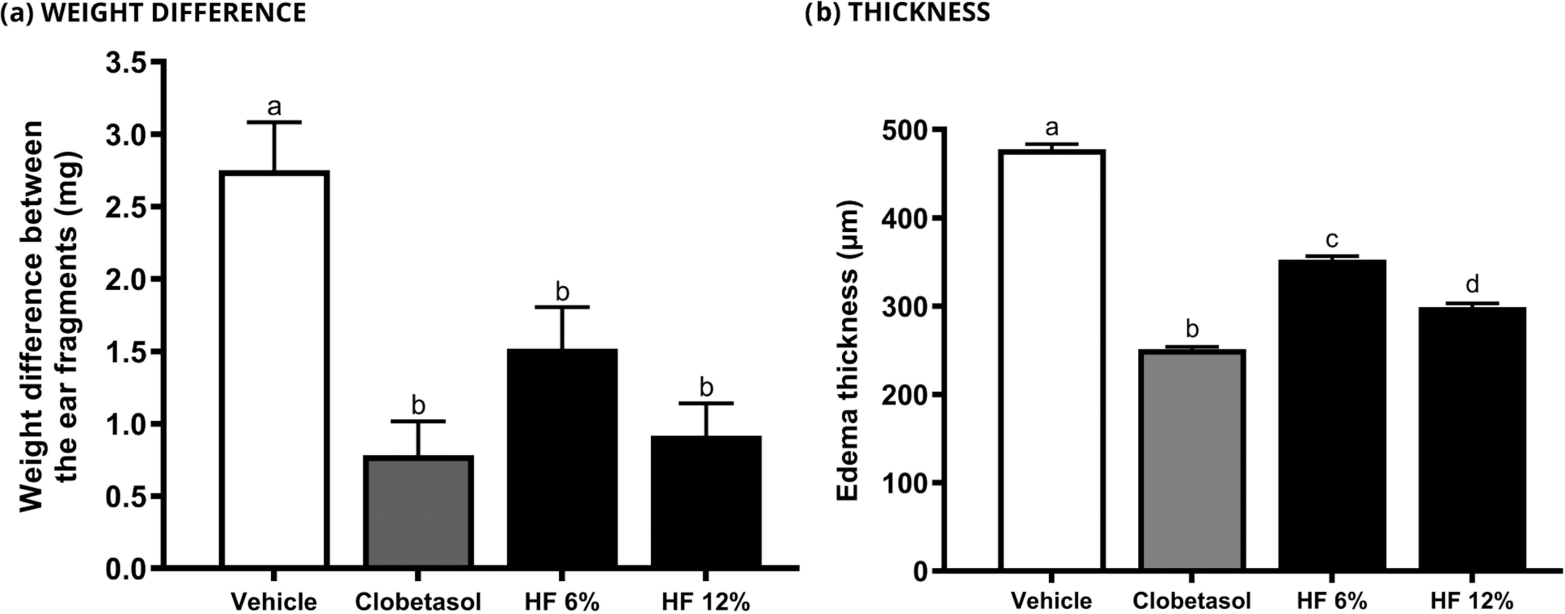
Effect of HF on the weight difference between ear fragments in the imiquimod-induced ear edema test (*n* = 8 animals). Negative control (vehicle - formulation), clobetasol 0.5 mg/g, HF 6 and 12% were topically applied for 10 days to investigate the antipsoriatic activity. Values in each column represent the mean ± s.e.m. of weight (mg) and thickness (μm) of the right ear edema fragments for each group. ANOVA, followed by the Newman-Keuls test. Means represented by equal letters indicate no statistical difference (*p* < 0.05).

A pharmaceutical formulation containing HF was used for the investigation in a psoriasis-like chronic inflammation. As shown in Figure 6a, the pharmaceutical prototype demonstrated remarkable antipsoriatic action *in vivo*. No statistical difference was found between the tested samples, including clobetasol (reference drug). On the other hand, the quantitative histological analysis revealed that HF 12% was more effective than HF 6%, but less effective than clobetasol (Figure 6b). It is important to mention that all treatments exhibited a significant difference in comparison to the vehicle group.

**Figure 7:**
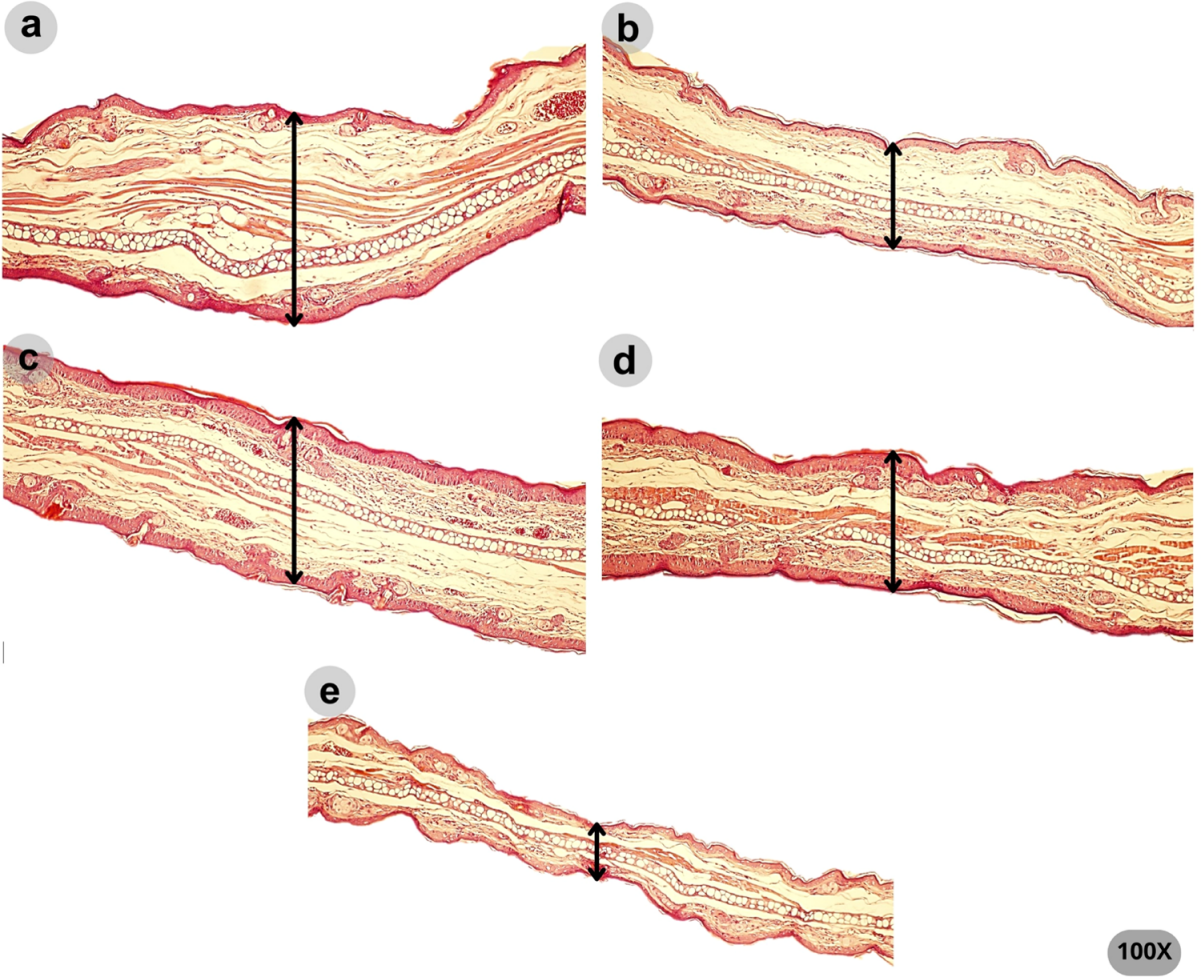
Representative photomicrographs (100× magnification) of the ear tissues on the last day of the imiquimod-induced ear edema test. The variation in edema thickness between groups is shown by the arrow. Means for each group were as follows: (a) vehicle (477.68 μm), (b) clobetasol (251.45 μm), (c) HF 6% (352.84 μm), (d) HF 12% (299.03 μm), and (e) left ear (186.17 μm).

**Figure 8:**
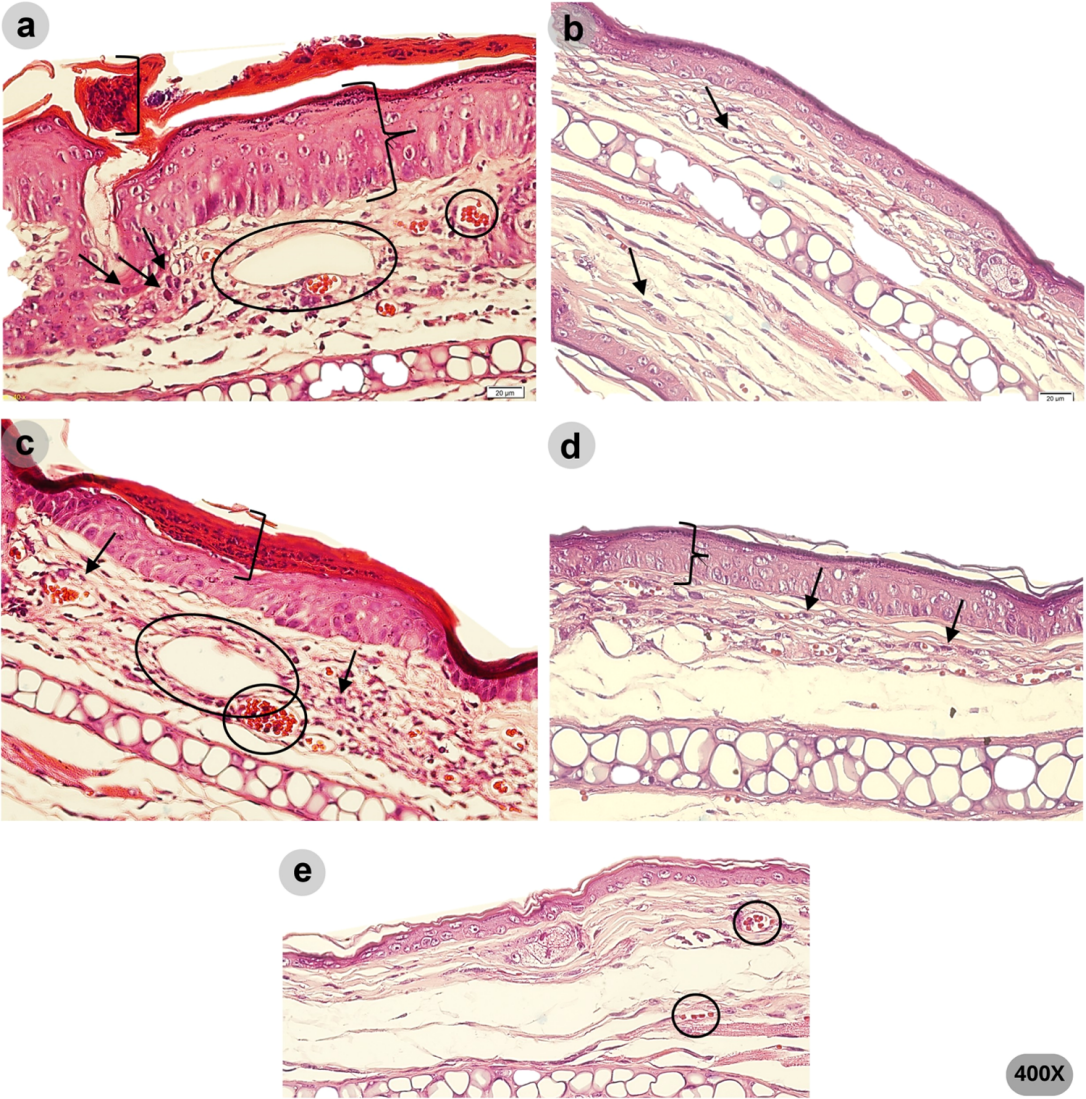
Representative photomicrographs (400× magnification) of the ear tissue on the last day of the imiquimod-induced ear edema test. The presence of nuclei in the corneal layer indicates parakeratosis (in brackets). Leukocyte infiltrate is indicated by arrows. Vasodilation was highlighted by a circle. Increased epidermal thickness (hyperkeratosis), typical of psoriasis lesions is shown in braces. (a) vehicle, (b) clobetasol, (c) HF 6%, (d) HF 12%, and (e) left ear.

The qualitative histopathological analysis is shown in Figures 7 and 8. Psoriasis-like morphological changes were detected in the ears treated with vehicle and HF 6%. These included parakeratosis, evidenced by the presence of nucleated keratinocytes in the *stratum corneum*, hyperkeratosis, inflammatory infiltrate, epidermal ridges, edema, and increased vascularity. In contrast, the ears treated with clobetasol and HF 12% showed less edema, and the typical morphological aspects of psoriatic inflammation were not prominent. In addition, the ears treated with clobetasol showed epidermis atrophy.

### 3.4 Phytochemical Evaluation

**Figure 9:**
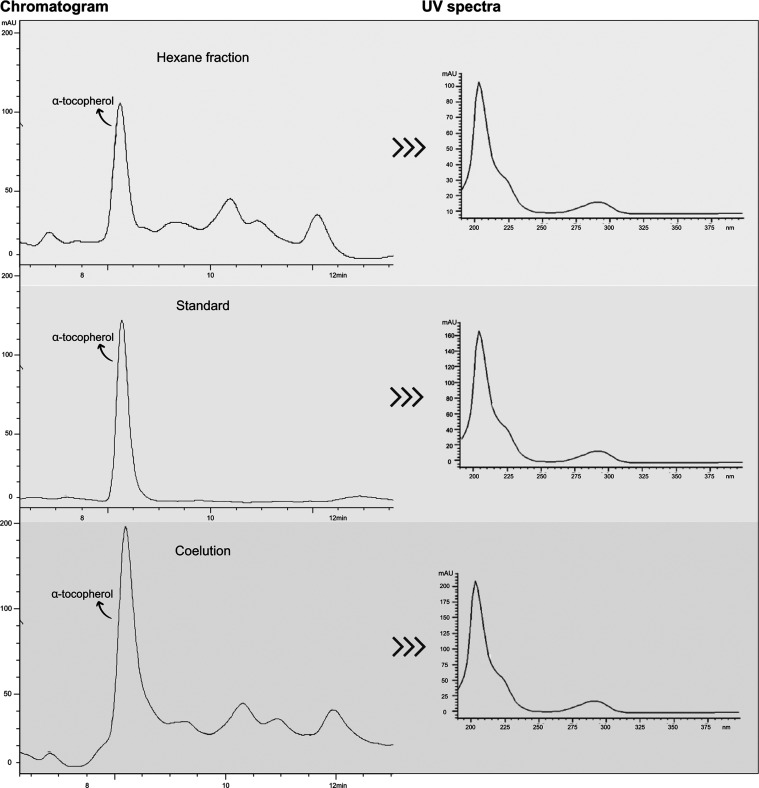
Chromatogram and UV spectra obtained by HPLC-UV analysis. The identification of α-tocopherol was carried out by comparing the UV spectra and retention time, and by coelution. Chromatographic conditions: Methanol and UHQ water (98:2, v/v) as mobile phase, at a flow rate of 1.3 mL/min. α-tocopherol standard at 25 μg/mL and PHEX at 1000 μg/mL were diluted in the mobile phase. The Injection volume was 20 μL, and the temperature was 25 °C. Agilent Eclipse Plus C18 column was used. UV detection was performed at 205 nm.

The HF phytochemical analysis revealed the presence of several classes of secondary metabolites. The TLC spray dyeing reagents detected the presence of steroids, phenols, flavonoids, and terpenes. Subsequently, tests to quantify the levels of steroids, phenols, and flavonoids were carried out, as shown in Table 2. Furthermore, the analysis by high-performance liquid chromatography (HPLC) revealed the presence of α-tocopherol, which content was 10.56 ± 0.97 mg/g of HF (Figure 9).

**Table 2:** Content of HF Steroids, Phenols, and Flavonoids Expressed in Milligram Equivalents of the Reference Substance per Gram

**steroid, phenol, and flavonoid content**	
	mg/g **de amostra**
steroids equivalents of β-sitosterol	491.68 ± 4.75
phenols equivalents of tannic acid	34.30 ± 4.96
flavonoids equivalents of quercetin	151.77 ± 6.66

## 4 Discussion

Reactive oxygen species (ROS) are known as harmful agents that damage various cellular components, leading to tissue damage. ROS are mainly produced by oxidative stress, which increases the risk of several diseases, genetic mutations, and inflammation.^31^ Antioxidant compounds act as potent allies to treat and prevent various disorders reducing the oxidative stress by neutralizing the free radicals derived from ROS.^32^ Indeed, many diseases are linked to the inflammatory process that often arises in response to pathogens or injury, which is associated with damage to DNA, lipids, and other macromolecules, strictly related to ROS accumulation.^33,34^

It is essential to perform different antioxidant assays for a tested sample due to the complexity of the *in vivo* oxidative stress. Each assay used in this study was designed to evaluate how a specific antioxidant inhibits different types of free radicals, so a complete assessment was conducted, providing a comprehensive view of *S. byzantina* effectiveness in protecting against damages induced by ROS, which are closely related to inflammatory mediation.^35^

First, the nitric oxide (NO) radical inhibition assay was accomplished. This radical plays distinct roles in regulating various functions within cells; however high levels of NO impair cellular functionality. The relationship between increased NOS (nitric oxide synthase) activity, which is the enzyme responsible for NO formation, and the development of inflammation is well-known. Immune cells, including macrophages and T cells, express NOS and generate NO in response to pathogens and several cytokines involved in inflammation, such as IL-1, TNF-α, and IFN-γ. These protein factors activate cell-surface receptors, inducing inflammatory pathways dependent on nuclear factor κB (NF-κB) and nuclear factor transducer and activator of transcription 1a (STAT-1a), which activates the promoter region of the NOS gene, stimulating NO synthesis. This free radical plays an important role in the immune host defense system and amplifies the inflammatory process. Generally, as NO levels increase the inflammatory process also increases. For this reason, some NOS inhibitors have been tested in clinical trials; however, none of them have been approved to date by regulatory agencies.^36^

The NO inhibitory assay was performed by monitoring NO generation after the spontaneous decomposition of sodium nitroprusside (NPS) in light exposure. Detection occurs indirectly, as NO is readily converted into nitrite due to oxygen action.^37^ As shown in Table 1, only EE and HF significantly inhibited the NO radical. For this reason, EE and HF were further investigated.

The β-carotene/linoleic acid system assay is frequently used to investigate the inhibition of lipid peroxidation by antioxidant compounds.^38^ The free radical generated from linoleic acid oxidation reacts with highly unsaturated β-carotene molecules. As β-carotene loses its double bonds due to oxidation, the color of this substance changes, which may be quantified by spectrophotometers.^39^ Therefore, the amount of degraded β-carotene was correlated with the antioxidant activity of the extracts.^40^ EE and HF exhibited a lipid peroxidation inhibition rate greater than 50% (Table 1). HF showed prominent action and a lower IC_50_ value, statistically different compared to EE.

Hydroxyl, alkyl, and peroxyl radicals are reactive oxygen species associated with lipid peroxidation.^41^ For this reason, the hydroxyl radical (^•^OH) inhibition assay based on the Fenton reaction was carried out. Although ^•^OH is often naturally produced during aerobic metabolism, it is the most reactive radical in the human body, so its inhibition is important to reduce damages associated with oxidative stress.^42^ EE and HF exhibited ^•^OH inhibition activity with no statistical difference; however, only HF showed no significant difference compared to the reference substance.

The ORAC test evaluates the ability of antioxidant compounds to inhibit both lipid peroxyl radicals (LOO^•^) and lipid alkoxyl radicals (LO^•^). It is widely used to predict the antioxidant activity in biological systems, including animals and plants.^43^ As shown in Figure 1, the fluorescence decreased over time for EE, HF, and the reference substance Trolox. No statistical difference between them was found for up to 30 min.

Considering that HF was the most promising antioxidant plant derivative, it was chosen for a more detailed and comprehensive investigation of its anti-inflammatory potential. *In vivo* tests were conducted using three different inflammatory agents: croton oil, phenol, and imiquimod. Croton oil contains a variety of compounds that activate protein kinase C and other inflammatory mediators, triggering local inflammation, edema, erythema, increased vascular permeability, leukocyte infiltration, and the release of histamine and serotonin.^44^ Croton oil also induces skin inflammation by activating an enzymatic cascade that includes phospholipase A_2_, leading to the release of arachidonic acid, prostaglandins, and platelet-activating factor. Corticosteroids, such as dexamethasone and clobetasol, have notable anti-inflammatory effects in this model, as well as COX and 5-LOX inhibitors, and leukotriene B_4_ antagonists.^45^

After the topical application of phenol, there is a disruption of keratinocyte membranes, leading to the release of pro-inflammatory molecules (IL-1α, IL-1β, IL-6, IL-8, and TNF-α). These cytokines, in turn, stimulate the release of several inflammatory mediators, including arachidonic acid metabolites and reactive oxygen species, which intensify the inflammatory process.^46,47^ The skin enzymes, including COX-2, LOX, and tyrosinase, generate a proper environment for the oxidation of one of the electrons in the phenol molecule, resulting in the formation of the phenoxyl radical.^48^ In this context, it is possible to conclude that compounds with antioxidant properties can play a significant role in mitigating the inflammation process induced by phenol.

Therefore, the HF ability to inhibit inflammation induced by croton oil and phenol suggests that it may interfere with crucial steps in the inflammatory cascade triggered by both phlogistic agents. Due to the activation of several different pathways, first, croton oil was used to screen HF at three different doses. HF 0.1 μg/20 μL and 0.5 μg/20 μL showed remarkable anti-inflammatory activity; however, HF 1.0 μg/20 μL showed no statistical difference compared to dexamethasone (Figure 2a). For this reason, HF 1.0 μg/20 μL was chosen for investigation by phenol-induced ear edema test. In this case, HF showed a significant response, statistically surpassing dexamethasone (Figure 2b). This HF response is probably due to its high antioxidant action, as phenol is a pro-inflammatory agent that more particularly triggers an inflammatory process associated with ROS production released during oxidative stress.^46^

Imiquimod (IMQ) is a therapeutic agent to treat genital and perianal warts; however, it is also an immune modulator. It is well-known that IMQ can trigger psoriasis-like skin lesions, which are characterized by several histomorphological changes, including epidermal hyperkeratosis, parakeratosis, intense inflammatory infiltrate, edema, and increased vascularity.^48,49^ IMQ action is mainly due to the interaction with TLR7 receptors in mice (in humans, with the TLR7 and TLR8 receptors). This interaction activates cellular signaling mediated by NFκB, which increases the production of several cytokines, including IL-12, promoting the differentiation of naive CD4^+^ T cells into IL-17-producing Th cells. IL-17, in turn, is a pro-inflammatory cytokine that stimulates fibroblasts, endothelial cells, macrophages, and epithelial cells to release several other inflammatory mediators, such as IL-6, TNF-α, nitric oxide synthase, metalloproteases and chemokines.^49,50^ In the past few years, there has been a significant improvement in psoriasis treatment, mainly due to the development of immunobiological drugs, such as TNF-α inhibitors, including infliximab, adalimumab, and etanercept, as well as ustekinumab, an IL-12/IL-23 inhibitor. Recently, IL-17 inhibitors were also introduced to the market, such as secukinumab, ixekizumab, brodalumab, and bimekizumab, in addition to IL-23 inhibitors, such as guselkumab, risankizumab, and tildrakizumab.^51^ However, access to immunobiological medicines is restricted due to their high cost and low industrial production. Thus, in response to the limitations of conventional pharmaceutical treatments, herbal medicine has emerged as an alternative therapy for psoriasis.^52^

Due to the promising acute HF anti-inflammatory activity, a pharmaceutical formulation was developed and evaluated by IMQ-induced ear edema test. HF 12% showed remarkable antipsoriatic potential, which opens up the perspective of this pharmaceutical formulation to be future used as an alternative or adjunct treatment for psoriasis (Figure 6). In addition, HF reduced the production of IL-12, IL-6, TNF-α, and NO (Figures 3 and 4) by macrophages, which are associated with the mechanisms of various inflammatory diseases. As previously mentioned, IL-12 plays a pivotal role in psoriasis pathophysiology.^51,53^ This cytokine is a heterodimer composed of the p35 subunit (IL-12p35) and the p40 subunit (IL-12p40). Both subunits bind to each other to form the bioactive IL-12 (IL-12p70).^54^ As IL-12p40 (evaluated in this study) is also part of the cytokine IL-23, which is involved in TH17 responses and psoriasis pathophysiology, the reduction of IL-12p40 levels after the treatment with HF could lead to IL-17 decreasing by IL-23 reduction.

It is noteworthy to mention that the doses and treatment regimen used in this study for HF alone and HF formulations was based on previous researches with some other plant species, such as *Cecropia pachystachya*, *Lacistema pubescens*, and *Pereskia aculeata*, which presented significant activities in this therapeutic scheme.^54−56^

The cell viabilities in cytotoxicity assays were maintained above 70% after the application of all HF concentrations tested, indicating no cytotoxicity. These results are in accordance with the guidelines established by ISO 10993-5. This finding is especially relevant when considering the topical application of HF, as fibroblast and keratinocytes are present in the skin, which supports HF promising potential as a safe and effective agent in dermatology.

Regarding the HF phytochemical analysis, TLC followed by spray dyeing reagents revealed the presence of at least four classes of secondary metabolites: steroids, phenols, flavonoids, and terpenes. Currently, the natural antioxidant, antimicrobial, anticarcinogenic, and anti-inflammatory properties of these compounds are areas of intensive research and application.^41^ In addition, some of them are recognized for stimulating the epithelialization process, promoting increased vessel formation, and regulating the response of inflammatory cytokines.^53^ Phytosterols exhibit a variety of biological effects, including hypocholesterolemic, antioxidant, anticancer, anti-inflammatory, and antibacterial activities.^57,58^ Particularly, campesterol, β-sitosterol, and stigmasterol have shown anti-inflammatory properties in several cell models.^38^ Phenolic compounds, such as flavonoids and phenolic acids, are widely recognized for their effective antioxidant activity and their ability to protect the human body against damage caused by free radicals by a variety of mechanisms of action.^59^ Studies report that there is a close link between the amount of total phenols and the antioxidant capacity of medicinal plants, including *S. byzantina*. Therefore, the results obtained regarding the levels of steroids, phenols, and flavonoids support *S. byzantina* anti-inflammatory activity reported by the present study.^60^

High-performance liquid chromatography (HPLC) is a relevant technique for identifying and quantifying secondary metabolites in plant derivatives, which is important for quality control purposes.^61^ There are reports on the presence of tocopherols in the genus *Stachys*, revealing an average of 13 to 15 mg of α-tocopherol per kg of essential oils. Furthermore, other compounds have been identified in *S. byzantina* K. Koch, including phenolic derivatives, such as caffeic acid, chlorogenic acid, and ferulic acid; flavonoids (rutin, apigenin), glycosides (verbascoside) and triterpenic/phytosterol derivatives (stigmasterol, β -sitosterol, and lawsaritol).^13−19^ In the study conducted by Ayaz & Eruygur (2022), two compounds were identified in the *Stachys* genus by HPLC. Ellagic acid and caffeic acid were the main components found in the methanolic extract, respectively. These compounds are known for their antioxidant and anti-inflammatory properties, as already reported in the scientific literature.^62^ In the present study, α-tocopherol was identified and quantified in HF by HPLC (10.56 mg/g), which properties are related to our findings, as α-tocopherol acts as a nonenzymatic antioxidant that, together with enzymatic antioxidants, such as glutathione peroxidase and superoxide dismutase, strongly contributes to the reduction of the oxidative stress, and consequently to the decreasing of the inflammatory process.^63^ Furthermore, α-tocopherol has been shown to interfere with several cellular responses, including those related to inflammation and apoptosis.^64^ In the study conducted by Yerlikaya et al. (2023), the importance of phytosterols is highlighted, which are classified into different groups based on their biological and structural functions and biosynthesis processes.^65^

## 5 Conclusions

This study shows that *S. byzantina* has promising potential to be used as an anti-inflammatory ingredient by the pharmaceutical industry. Particularly, the hexane fraction revealed promising potential to be used for skin inflammation, including psoriasis. HF mode of action was associated with the inhibition of pro-inflammatory cytokines released after the tissue damage, including IL-12, which is relevant in psoriasis physiopathology, the prevention of ROS formation, the ability to neutralize free radicals, or all of these processes together. Furthermore, cytotoxicity assays demonstrated that HF is probably safe to be applied on the skin.

## References

[ref1] Gallegos-Alcalá P.; Jiménez M.; Cervantes-garcía D.; Córdova-dávalos L. E.; Gonzalez-curiel I.; Salinas A. Glycomacropeptide Protects against Inflammation and Oxidative Stress and Promotes Wound Healing in an Atopic Dermatitis Model of Human Keratinocytes. Foods 2023, 12, 1932 10.3390/foods12101932. 37238750 PMC10217156

[ref2] Kabashima K.; Honda T.; Ginhoux F.; Egawa G. The immunological anatomy of the skin. Nat. Rev. Imunol. 2019, 19, 19–30. 10.1038/s41577-018-0084-5. 30429578

[ref3] Wagener F.A.D.T.G.; Carels C. E.; Lundvig D. M. S. Targeting the redox balance in inflammatory skin conditions. Int. J. Mol. Sci. 2013, 14, 9126–9167. 10.3390/ijms14059126. 23624605 PMC3676777

[ref4] Khan A. Q.; Agha M. V.; Sultan M. K.; Sheikhan A.; Younis S. M.; Tamimi M. A.; Alam M.; Ahma A.; Uddin S.; Buddenkotte J.; Steinhoff M. Targeting deregulated oxidative stress in skin inflammatory diseases: An update on clinical importance. Biomed. Pharmacother. 2022, 154, 113601 10.1016/j.biopha.2022.113601. 36049315

[ref5] Poetker D. M.; Reh D. D. A comprehensive review of the adverse effects of systemic corticosteroids. Otolaryngol. Clin. North Am. 2010, 43, 753–768. 10.1016/j.otc.2010.04.003. 20599080

[ref6] Global Market Research Reports & Consulting, 2024. The Business Research Company. https://www.thebusinessresearchcompany.com/ (accessed March 22, 2024).

[ref7] Fattahi Ardakani M.; Salahshouri A.; Sotoudeh A.; Fard M. R.; Dashti S.; Chenari H. A.; Baumann S. L. A Study of the Use of Medicinal Plants by Persons With Type 2 Diabetes in Iran. Nurs. Sci. Q. 2024, 37, 168–172. 10.1177/08943184231224454. 38491885

[ref8] Agidew M. G. Phytochemical analysis of some selected traditional medicinal plants in Ethiopia. Bull. Natl. Res. Cent. 2022, 46, 87 10.1186/s42269-022-00770-8.

[ref9] Cakilcioglu U.; Khatun S.; Turkoglu I.; Hayta S. Ethnopharmacological survey of medicinal plants in Maden (Elazig-Turkey). J. Ethnopharmacol. 2011, 137, 469–486. 10.1016/j.jep.2011.05.046. 21704144

[ref10] Tomou E. M.; Barda C.; Skaltsa H. Genus Stachys: A Review of Traditional Uses, Phytochemistry and Bioactivity. Medicines 2020, 7, 63 10.3390/medicines7100063. 33003416 PMC7601302

[ref11] Messias M. C. T. B.; Menegatto M. F.; Prado A. C. C.; Santos B. R.; Guimarães M. F. M. Uso popular de plantas medicinais e perfil socioeconômico dos usuários: um estudo em área urbana em Ouro Preto, MG, Brasil. Rev. Bras. Plant. Med. 2015, 17, 76–104. 10.1590/1983-084X/12_139.

[ref12] Stegăruş D. I.; Lengyel E.; Apostolescu G. F.; Botoran O. R.; Tanase C. Phytochemical Analysis and Biological Activity of Three Stachys Species (Lamiaceae) from Romania. Plants 2021, 10, 2710 10.3390/plants10122710. 34961181 PMC8709469

[ref13] Aminfar P.; Abtahi M.; Parastar H. Gas chromatographic fingerprint analysis of secondary metabolites of Stachys lanata (*Stachys byzantine* C. Koch) combined with antioxidant activity modeling using multivariate chemometric methods. J. Chromatogr A 2019, 1602, 432–440. 10.1016/j.chroma.2019.06.002. 31230874

[ref14] Sarikurkcu C.; Kocak M. S.; Uren M. C.; Calapoğlu M. Potential sources for the management global health problems and oxidative stress: *Stachys byzantina* and *S. iberica* subsp. iberica var. densipilosa. Eur. J. Integr. Med. 2015, 540, 631–637. 10.1016/j.eujim.2016.04.010.

[ref15] Asnaashari S.; Delazar A.; Alipour S. S.; Nahar L.; et al. Chemical composition, free-radical-scavenging and insecticidal activities of the aerial parts of *Stachys byzantina*. Arch. Biol. Sci. 2010, 62, 653–662. 10.2298/ABS1003653A.

[ref16] Khanavi M.; Sharifzadeh M.; Hadjiakhoondi A.; Shafiee A. Phytochemical investigation and anti-inflammatory activity of aerial parts of *Stachys byzantina* C. Koch. J. Ethnopharmacol. 2005, 97, 463–468. 10.1016/j.jep.2004.11.037. 15740882

[ref17] Ebrahimzadeh M. A.; Nabavi S. M.; Nabavi S. F.; Bahramian F.; Bekhradnia A. R. Antioxidant and free radical scavenging activity *of**H. officinalis**L. Var. Angustifolius*, *V. odorata*, *B. hyrcana**and**C. speciosum*. Pak. J. Pharm. Sci. 2010, 23, 29–34. 10.5897/jmpr2017.6421. 20067863

[ref18] Marco G. J. A. A rapid method for evaluation of antioxidants. J. Am. Oil Chem. Soc. 1968, 45, 594–598. 10.1007/BF02668958.

[ref19] Gligorovski S.; Strekowski R.; Barbati S.; Vione D. Environmental implications of hydroxyl radicals (HO^•^). Chem. Rev. 2015, 115, 13051–13092. 10.1021/cr500310b. 26630000

[ref20] Ou B.; Hampsch-Woodill M.; Prior R. Development and Validation of an Improved Oxygen Radical Absorbance Capacity Assay Using Fluorescein as the Fluorescent Probe. J. Agric. Food Chem. 2001, 49, 4619–4626. 10.1021/jf010586o. 11599998

[ref21] Riss T. L.; Moravec R. A.; Niles A. L.; Duellman S.; Benink H.Á.; Worzella T. J.; Minor L.; Markossian S.; Sittampalam G. S.; Grossman A.; Brimacombe K.; Arkin M.; Auld D.; Austin C.; Baell J.; Caavaeiro J.M.M.C.; Chung T. D. Y.; Coussens N. P.; Dahlin J. L.; Devanaryan V.; Foley T. L.; Glicksman M.; Hall M. D.; Haas J. V.; Hoare S. R. J.; Inglese J.; Iversen P. W.; Kahl S. D.; Kales S. C.; Kirshner S.; Lal-nag M.; Li Z.; Mcgee J.; Mcmanus O.; Riss T.; Saradjian P.; Jr O. J. T.; Weidner J. R.; Wildey M. J.; Xia M.; Xu X. Cell Viability Assays. In Eli Lilly & Company and the National Center for Advancing Translational Sciences, editors. Assay Guidance Manual [Internet]; E-Publishing Inc: Bethesda, 2013.

[ref22] Guevara I.; Iwanejko J.; Dembińska-Kieć A.; Pankiewicz J.; Wanat A.; Anna P.; Gołabek I.; Bartuś S.; Malczewska-Malec M.; Szczudlik A. Determination of nitrite/nitrate in human biological material by the simple Griess reaction. Clin. Chim. Acta 1998, 274, 177–188. 10.1016/S0009-8981(98)00060-6. 9694586

[ref23] Schiantarelli P.; Cadel S.; Acerbi D.; Pavesi L. Antiiflamatory activity and bioavailability of percutaneous piroxican. Arzneim.-Forsch./Drug Res. 1982, 32, 230–235. 10.1590/S1516-93322008000300013. 7200781

[ref24] Gábor M. Mouse Ear Inflammation Models and Their Pharmacological Applications; Akadémiai Kiadó: Budapeste, 2000.

[ref25] Sun Y.; Zhang J.; Huo R.; Zhai T.; Li H.; Wu P.; Zhu X.; Zhou Z.; Shen B.; Li N. Paeoniflorin inhibits skin lesions in imiquimod-induced psoriasis-like mice by downregulating inflammation. Int. Immunopharmacol 2015, 24, 392–399. 10.1016/j.intimp.2014.12.032. 25576402

[ref26] Bladt S.; Wagner H. Plant drug analysis: A thin layer chromatography atlas; Springer-Verlag: Berlin, 1984.

[ref27] Pedrosa A. M.; Castro W. V.; Castro A. H. F.; Duarte-Almeida J. M. Validated spectrophotometric method for quantification of total triterpenes in plant matrices. DARU 2020, 28, 281–286. 10.1007/s40199-020-00342-z. 32285314 PMC7214563

[ref28] Singleton V. L.; Rossi J. A. Colorimetry of total phenolics with phosphor- molybdic phosphotungstic acid reagents. Am. J. Enol. Vitic. 1965, 16, 144–158. 10.5344/ajev.1965.16.3.144.

[ref29] Hao Z.; Liang L.; Liu H.; Yan Y.; Zhang Y. Exploring the Extraction Methods of Phenolic Compounds in Daylily (*Hemerocallis citrina* Baroni) and Its Antioxidant Activity. Molecules 2022, 27, 2964 10.3390/molecules27092964. 35566310 PMC9101449

[ref30] Delgado-Zamarreño M. M.; Fernández-Prieto C.; Bustamante-Rangel M.; Pérez-Martín L. Determination of tocopherols and sitosterols in seeds and nuts by QuEChERS-liquid chromatography. Food Chem. 2016, 192, 825–830. 10.1016/j.foodchem.2015.07.083. 26304416

[ref31] Djedjibegovic J.; Marjanovic A.; Panieri E.; Saso L. Ellagic Acid-Derived Urolithins as Modulators of Oxidative Stress. Oxid. Med. Cell. 2020, 2020, 1–15. 10.1155/2020/5194508. PMC7407063 32774676

[ref32] Davies M. J. Quantification and Mechanisms of Oxidative Stress in Chronic Disease. Proceedings 2019, 11, 1–18. 10.3390/proceedings2019011018.

[ref33] Greten F. R.; Grivennikov S. I. Inflammation and Cancer: Triggers, Mechanisms, and Consequences. Immunity 2019, 51, 27–41. 10.1016/j.immuni.2019.06.025. 31315034 PMC6831096

[ref34] Lopez-corona A. V.; Valencia-espinosa I.; González-sánchez F. A.; Sánchez-lópez A. L.; Garcia-amezquita L. E.; Garcia-varela R. Antioxidant, Anti-Inflammatory and Cytotoxic Activity of Phenolic Compound Family Extracted from Raspberries (Rubusidaeus): A General Review. Antioxidants 2022, 11, 1192–1212. 10.3390/antiox11061192. 35740089 PMC9230908

[ref35] Świerczek A.; Jusko W. J. Pharmacokinetic/Pharmacodynamic Modeling of Dexamethasone Anti-Inflammatory and Immunomodulatory Effects in LPS-Challenged Rats: A Model for Cytokine Release Syndrome. J. Pharmacol. Exp. Ther. 2023, 384, 455–472. 10.1124/jpet.122.001477. 36631280 PMC9976795

[ref36] Lundberg J. O.; Weitzberg E. Nitric oxide signaling in health and disease. Cell 2022, 185, 2853–2878. 10.1016/j.cell.2022.06.010. 35931019

[ref37] Green L. C.; Wagner D. A.; Glogowski J.; Skipper P. L.; Wishnok J. S.; Tannenbaum S. R. Analysis of nitrate, nitrite and nitrate in biological fluids. Anal. Biochem. 1982, 126, 131–138. 10.1016/0003-2697(82)90118-X. 7181105

[ref38] Zhang Y.; Cai P.; Cheng C.; Zhang Y. A Brief Review of Phenolic Compounds Identified from Plants: Their Extraction, Analysis, and Biological Activity Nat. Prod. Commun. 2022, 17, 10.1177/1934578X211069721.

[ref39] Gillani F.; Amiri Z. R.; Kenari R. E. Assay of Antioxidant Activity and Bioactive Compounds of *Cornelian Cherry* (Cornus mas L.) Fruit Extracts Obtained by Green Extraction Methods: Ultrasound-Assisted, Supercritical Fluid, and Subcritical Water Extraction. Pharm. Chem. J. 2022, 56, 692–699. 10.1007/s11094-022-02696-x.

[ref40] Ghasemzadeh A.; Jaafar H. Z. E.; Juraimi A. S.; Tayebi-meigooni A. Comparative evaluation of different extraction techniques and solvents for the assay of phytochemicals and antioxidant activity of hashemi rice bran. Molecules 2015, 20, 10822–10838. 10.3390/molecules200610822. 26111171 PMC6272729

[ref41] Zhang S.; Lia H.; Lid M.; Chenc G.; Maorcid Y.; Wanga Y.; Chena J. Construction of ferulic acid modified porous starchesters for improving the antioxidant capacity. RSC Adv. 2022, 12, 4253–4262. 10.1039/D1RA08172A. 35425409 PMC8981049

[ref42] Sihag S.; Pal A.; Ravikant; Saharan V. Antioxidant properties and free radicals scavenging activities of pomegranate (*Punica granatum* L.) peels: An *in-vitro* study. Biocatal. Agric. Biotechnol. 2022, 42, 102368 10.1016/j.bcab.2022.102368.

[ref43] Munteanu I. G.; Apetrei C. Analytical Methods Used in Determining Antioxidant Activity: A Review. Int. J. Mol. Sci. 2021, 22, 33–80. 10.3390/ijms22073380. PMC8037236 33806141

[ref44] Pinto N. d. C. C.; Maciel M. S. F.; Rezende N. S.; Duque A. P. N.; Mendes R. F.; Silva J. B.; Evangelista M. R.; Monteiro L. C.; Silva J. M.; Costa J. C.; Scio E. Preclinical studies indicate that INFLATIV, an herbal medicine cream containing *Pereskia aculeata*, presents potential to be marketed as a topical anti-inflammatory agent and as adjuvant in psoriasis therapy. J. Pharm. Pharmacol. 2020, 72, 1933–1945. 10.1111/jphp.13357. 32846458

[ref45] Ferreira E. A.; Queiroz L. S.; Facchini G. F. S.; Guedes M. C. M. R.; Macedo G. C.; Sousa E. V.; Filho A. A. S. *Baccharis dracunculifolia* DC (Asteraceae) Root Extractand Its Triterpene *Baccharis* Oxide Display Topical Anti-Inflammatory Effect son Different Mice Ear Edema Models. Evid. Based Complement Alternat. Med. 2023, 2023, 9923941 10.1155/2023/9923941. 37275573 PMC10234725

[ref46] Murray A. R.; Kisin E.; Castranova V.; Kommineni C.; Gunther M. R.; Shvedova A. A. Phenol-induced in vivo oxidative stress in skin: evidence for enhanced free radical generation, thiol oxidation, and antioxidant depletion. Chem. Res. Toxicol. 2007, 20, 1769–1777. 10.1021/tx700201z. 17922553

[ref47] Bomfim R. R.; Oliveira J. P.; Abreu F. F.; Oliveira A. S.; Correa C. B.; Jesus E.; Alves P. B.; Santos M. B.; Grespan R.; Camargo E. A. Topical Anti-inflammatory Effect of Annonamuricata (graviola) Seed Oil. Rev. Bras. Farmacogn. 2023, 33, 95–105. 10.1007/s43450-022-00292-4.

[ref48] Yadanar H. O.; Thandar L. S.; Lwin S. M. M.; Thu A. L.; Myint K. S.; Rattanakanokchai S.; Sothornwit J.; Aue-aungkul A.; Pattanittum P.; Ngamjarus C.; Tin K. N.; Show K. L.; Jampatong N.; Lumbiganon P. Topical imiquimod cream for the treatment of cervical intraepithelial neoplasia. Cochrane Database Syst. Rev. 2024, 2024 (3), CD015867 10.1002/14651858.CD015867.

[ref49] Flutter B.; Nestle F. O. TLRs to cytokines: mechanisms insights from imiquimod mouse model of psoriasis. Eur. J. Immunol. 2013, 43, 3138–3146. 10.1002/eji.201343801. 24254490

[ref50] Iwakura Y.; Ishigame H. The IL-23/IL-17 axis in inflammation. J. Clin Invest. 2006, 116, 1218–1222. 10.1172/JCI28508. 16670765 PMC1451213

[ref51] Riel C. A. M.; Michielsens C. A. J.; Muijen M. E.; Van der Schoot L. S.; Van den Reek J. M. P. A.; Jong E. M. G. J. Dose reduction of biologics in patients with plaque psoriasis: a review. Front. Pharmacol. 2024, 15, 1369805 10.3389/fphar.2024.1369805. 38606178 PMC11007084

[ref52] Paul T.; Kumar J. Standardization of Herbal Medicines for Lifestyle Diseases. In Role of Herbal Medicine 2023; Vol. 2023, pp 545–556 10.1007/978-981-99-7703-1_27.

[ref53] Mushtaq R.; Akram A.; Mushtaq R.; Khwaja S.; Ahmed S. The role of inflammatory markers following Ramadan Fasting. Pak J. Med. Sci. 2018, 35, 77–81. 10.12669/pjms.35.1.95. PMC6408667 30881400

[ref54] Pacheco N. R.; Pinto N. C. C.; Silva J. M.; Mendes R. F.; Costa J. C.; Aragão D. M. O.; Castañon M. C. M. N.; Scio E. *Cecropia pachystachya*: a species with expressive in vivo topical anti-inflammatory and *in vitro* antioxidant effects. BioMed Res. Int. 2014, 2014, 1–10. 10.1155/2014/301294. PMC4022158 24877079

[ref55] Pinto N. d. C. C.; Maciel M. C. F.; Rezende N. S.; Duque A. P. N.; Mendes R. F.; Silva J. B.; Evangelista M. R.; Monteiro L. C.; Silva J. M.; Costa J. C.; Scio E. Preclinical studies indicate that INFLATIV, an herbal medicine cream containing *Pereskia aculeata*, presents potential to be marketed as a topical anti-inflammatory agent and as adjuvant in psoriasis therapy. J. Pharm. Pharmacol. 2020, 72, 1933–1945. 10.1111/jphp.13357. 32846458

[ref56] Silva J. M.; Conegundes J. L. M.; Pinto N. C. C.; Mendes R. F.; Castañon M. C. M. N.; Scio E. Comparative analysis of Lacistema pubescens and dexamethasone on topical treatment of skin inflammation in a chronic disease model and side effects. J. Pharm. Pharmacol. 2018, 70 (4), 576–582. 10.1111/jphp.12886. 29441584

[ref57] Mssillou I.; Bakour M.; Slighoua M.; Laaroussi H.; Saghrouchni H.; Amrati F. A.; Lyoussi B.; Derwich E. Investigation on wound healing effect of Mediterranean medicinal plants and some related phenolic compounds: A review. J. Ethnopharmacol. 2022, 298, 115663 10.1016/j.jep.2022.115663. 36038091

[ref58] Salehi B.; Quispe C.; Sharifi-Rad J.; Cruz-Martins N.; Nigam M.; Mishra A. P.; Konoralov D. A.; Orobinskaya V.; Abu-Reidah I.; Zam W.; Sharopov F.; Venneri T.; Capasso R.; Kukula-Koch W.; Wawruszak A.; Koch W. Phytosterols: from preclinical evidence to potential clinical applications. Front. Pharmacol. 2021, 11, 599959 10.3389/fphar.2020.599959. 33519459 PMC7841260

[ref59] Zeghbib W.; Boudjouan F.; Bachir-bey M. Optimization of phenolic compounds recovery and antioxidant activity evaluation from Opuntia ficus indica using response surface methodology. J. Food Meas Charact. 2022, 16, 1354–1366. 10.1007/s11694-021-01241-w.

[ref60] Bilušić Vundać V.; Brantner A. H.; Plazibat M. Content of polyphenolic constituents and antioxidant activity of some *Stachys* taxa. Food Chem. 2007, 104, 1277–1281. 10.1016/j.foodchem.2007.01.036.

[ref61] Nadal J. M.; Toledo M. G.; Pupo Y. M.; Paula J. P.; Farago P. V.; Zanin S. M. W. A Stability-Indicating HPLC-DAD Method for Determination of Ferulic Acid into Microparticles: Development, Validation, Forced Degradation, and Encapsulation Efficiency. J. Anal Methods Chem. 2015, 2015, 286812 10.1155/2015/286812. 26075139 PMC4446543

[ref62] Ayaz F.; Eruygur N. Investigation of enzyme inhibition potentials, and antioxidative properties of the extracts of endemic *Stachys bombycina* Boiss. Istanbul J. Pharm. 2022, 52, 318–323. 10.26650/IstanbulJPharm.2022.1075293.

[ref63] Lee A. R. Y. B.; Tariq A.; Lau G.; Tok N. W. K.; Tam W. W. S.; Ho C. S. H. Vitamin E, Alpha-Tocopherol, and Its Effects on Depression and Anxiety: A Systematic Review and Meta-Analysis. Nutrients 2022, 14, 656–674. 10.3390/nu14030656. 35277015 PMC8840247

[ref64] Liao S.; Omage S. O.; Börmel L.; Kluge S.; Schubert M.; Wallert M.; Lorkowski A. Vitamin E and Metabolic Health: Relevance of Interactions with Other Micronutrients. Antioxidants 2022, 11, 1785–1816. 10.3390/antiox11091785. 36139859 PMC9495493

[ref65] Yerlikaya P. O.; Arisan E. D.; Mehdizadehtapeh L.; Uysal-Onganer P.; Coker-Gurkan A. The use of plant steroids in viral disease treatments: current status and future perspectives. Eur. J. Biol. 2023, 82, 86–94. 10.26650/EurJBiol.2023.1130357.

